# Melatonin in Retinal Physiology and Pathology: The Case of Age-Related Macular Degeneration

**DOI:** 10.1155/2016/6819736

**Published:** 2016-09-05

**Authors:** Janusz Blasiak, Russel J. Reiter, Kai Kaarniranta

**Affiliations:** ^1^Department of Molecular Genetics, University of Lodz, Pomorska 141/143, 90-236 Lodz, Poland; ^2^Department of Cellular & Structural Biology, University of Texas Health Science Center, San Antonio, TX 78229-3900, USA; ^3^Department of Ophthalmology, University of Eastern Finland, 70211 Kuopio, Finland; ^4^Department of Ophthalmology, Kuopio University Hospital, 70029 Kuopio, Finland

## Abstract

Melatonin, an indoleamine, is synthesized mainly in the pineal gland in a circadian fashion, but it is produced in many other organs, including the retina, which seems to be especially important as the eye is a primary recipient of circadian signals. Melatonin displays strong antioxidative properties, which predispose it to play a protective role in many human pathologies associated with oxidative stress, including premature aging and degenerative disease. Therefore, melatonin may play a role in age-related macular degeneration (AMD), a disease affecting photoreceptors, and retinal pigment epithelium (RPE) with an established role of oxidative stress in its pathogenesis. Several studies have shown that melatonin could exert the protective effect against damage to RPE cells evoked by reactive oxygen species (ROS), but it has also been reported to increase ROS-induced damage to photoreceptors and RPE. Melatonin behaves like synthetic mitochondria-targeted antioxidants, which concentrate in mitochondria at relatively high levels; thus, melatonin may prevent mitochondrial damage in AMD. The retina contains telomerase, an enzyme implicated in maintaining the length of telomeres, and oxidative stress inhibits telomere synthesis, while melatonin overcomes this effect. These features support considering melatonin as a preventive and therapeutic agent in the treatment of AMD.

## 1. Introduction

Melatonin,* N*-acetyl-5-methoxytryptamine, indole found in virtually all forms of life, from bacteria to humans, is a highly potent biomolecule with many functions in biological systems, the vast majority of which are beneficial [1–5]. Moreover, melatonin has a high safety profile [6]. Melatonin functions are either receptor-dependent or receptor-independent [7]. The former include circadian rhythm control and sleep regulation, whereas the latter include the detoxification of reactive oxygen species (ROS) and other reactive molecules. Due to this latter function, melatonin plays a role in cellular defense against oxidative stress [8–10].

Melatonin is released from the pineal gland in a circadian fashion, which is controlled by suprachiasmatic nuclei (SCN) [11]. However, many other tissues and organs include the retina, lens, gastrointestinal tract, and skin synthesize melatonin [12, 13]. Melatonin's antioxidant properties combined with its circadian regulatory properties may be especially important in ophthalmology as the eye is a primary receptor of rhythmic light/dark signals sent from retinal ganglion cells to SCN; also the retina is a region of intense blood flow resulting in high oxygen levels in this organ. A single-pulse light stimulation resulted in an over 50% increase in the retinal blood flow [14, 15]. These high concentration of oxygen requires a strict redox control to prevent oxidative stress. Also light that reaches the retina generates ROS that contribute to oxidative stress [16, 17]. Moreover, retinal cells and retinal pigment epithelium (RPE) cells are rich in mitochondria, which are a major source of ROS produced by the electron transport chain (ETC) [18].

**Figure 1:**
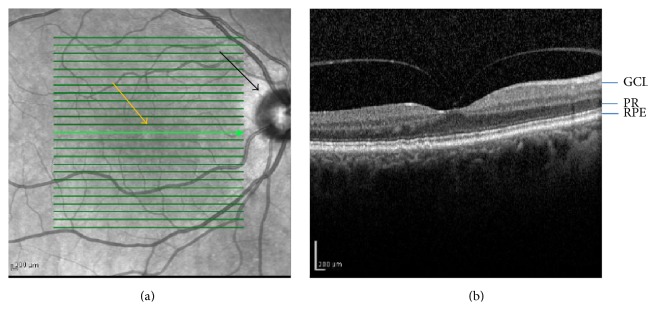
(a) Photograph of the fundus of the central retina. Yellow arrow indicates fovea in central macula and black arrow identifies the optic nerve head. (b) Optical coherent tomography image from a normal retina. GCL, ganglion cells; PR, photoreceptors; RPE, retinal pigment epithelium.

Age-related macular degeneration (AMD) is an eye disease characterized by degenerative changes in the central retina known as the macula [19] (Figure 1(a)). It is a complex disease, whose pathogenesis results from the interaction between many genetic and environmental factors. The most important genetic factor in AMD pathogenesis seems to be variations in the complement factor H (CFH).

**Figure 2:**
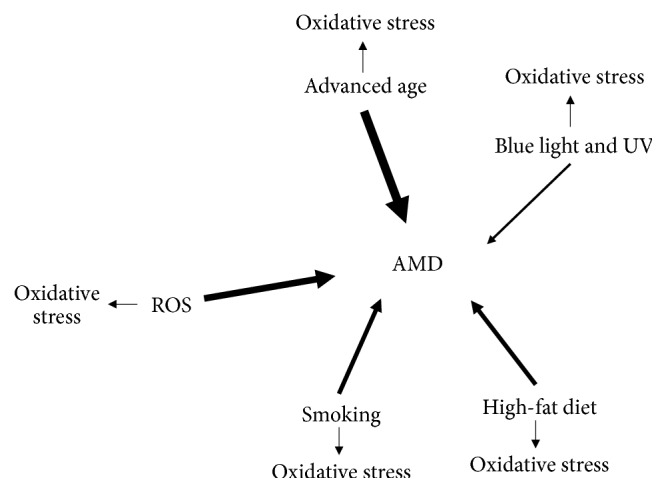
Major AMD environmental risk factors. The thickness of arrows corresponds to a putative significance of a particular factor. These factors are associated with increased ROS production which contributes to oxidative stress. ROS, reactive oxygen species.

Major environmental risk factors for AMD are age, smoking, oxidative stress, high-fat diet, light, and UV (Figure 2) [20]; all of these increase production of highly reactive and toxic ROS. Thus, oxidative stress is a consequence of all the main environmental risk factors. Moreover, the influence of variations in the CHF can be modulated by oxidative stress. Female gender, Caucasian origin, and light iris are frequently mentioned as minor AMD risk factors.

The nature of the interaction between genetic and environmental factors is mostly unknown, but oxidative stress is unequivocally associated with the induction and development of AMD. The fundamental question as whether oxidative stress is a casual factor for AMD or merely accompanies disease progression remains unanswered, however. This problem is additionally complicated by the age-dependent accumulation of lipofuscin in highly pigmented postmitotic RPE cells. The RPE-derived pigment protects the neuroretina from the excess of light exposure [21]. Normal and young RPE cells effectively degrade proteins in lysosomes, but this process is estimated to be impaired in aging cells which ultimately lead to the detrimental accumulation of lipofuscin in lysosomes [22]. Lipofuscin can be photooxidized yielding ROS. Therefore, the retina is prone to oxidative stress and its important functions of receiving light signals, contributing to circadian regulation, and releasing melatonin in a circadian fashion are negatively impacted in AMD. The implication is that melatonin likely plays a role in AMD pathogenesis. In this review we summarize some aspects of mutual relationships between melatonin and retinal physiology/pathology to document some potential preventive and therapeutic potential of melatonin in AMD.

## 2. The Retina as a Light-Sensitive Ocular Clock

The retina is an essential part of the back of the eye; it consists of layer of cells about 0.5 mm in thickness (Figure 1). It includes ganglion cells, located innermost, and the photosensors (photoreceptors), the rods and cones, lying closest to the RPE (Figure 1(b)). The human retina contains three layers of nerve cells and two regions of synapses. Three types of glial cells are found in the human retina: Muller cells, astroglia, and microglia. Macula (*macula lutea*) is a yellowish area located near the center of the retina, which is responsible for the central vision and the visual acuity (Figure 1). It has a specific structure in its center, called the fovea, which contains exclusively cones, specialized nerve cells associated with color vision and perception of tiny objects.

As the nervous system exhibits circadian rhythm and since the retina is considered as a part of the brain and is influenced by the day/night cycle, it is not surprising that it has its own retinal clock. In general, there are several genes, collectively known as “clock genes,” the expression of which is necessary for circadian rhythmicity [23]. The products of these genes regulate transcription of “clock controlled” genes. More precisely, the molecular clock in the retina is controlled by two feedback-loops: one is positive and consists of the transcription factors BMAL1 and CLOCK, activating in a heterodimeric form transcription of the PERIOD and CRYPTOCHROME genes. The second is a negative loop where cytoplasmic PERIOD and CRYPTOCHROME form a heterodimer, which enters the nucleus and inhibits transactivation of the promoters of their own genes. The retinal circadian rhythm is independent of the rest of nervous system as it was demonstrated by the release of melatonin in a circadian fashion by isolated retinal cells [24]. The expression of the* CLOCK* gene was reported not to be needed for molecular rhythms in the mouse SCN [25]. The mouse retina differs from its bird and amphibian counterparts, where the photoreceptors are internal circadian oscillators involved in rhythmic release of melatonin [26–28].

At least some of the clock controlled genes have circadian E boxes in their promotors. The nucleotide sequence of the E box contains the core motif 5′-CACGTG clock, bound periodically by the CLOCK/BMAL1 heterodimer [29]. It is not the only interaction important for the circadian regulation in the retina as several posttranslational modifications, including acetylation, phosphorylation, sumoylation, and ubiquitination, were shown to have roles in this phenomenon as well [30, 31]. Sustained circadian rhythms in the retina were demonstrated in Muller glia cells which express canonical circadian clock genes [32].

The retinal circadian clock influences many processes occurring in the retina, including melatonin secretion, dopamine synthesis, visual sensitivity, extracellular pH, and intraocular pressure as well as some electroretinogramic responses [33]. The clock is also involved in some pathological processes in the retina including degeneration of photoreceptors upon interaction with light [34, 35]. In spite of its influential effects on the function of the retina in mammals, the organization and origin of the retinal clock are not completely known.

## 3. Melatonin in the Eye

Although melatonin was first identified as being synthesized in the pineal gland, it is also produced in many other organs [12, 13], where it is believed to have important local functions as paracoid or autocoid [36]. In the retina, melatonin is released mainly by photoreceptor cells but can be also produced by some other cells in pathological conditions [37]. It was shown that the rat retina continued producing melatonin in a circadian manner even after pinealectomy [3]. Thus, retinal melatonin production is under the control of retinal circadian clock, resulting in its high concentration in the night and low level during the day, although its amount is small compared to its pineal counterpart [38, 39].

As in the pineal gland, nocturnal melatonin synthesis in the retinal photoreceptors of mammals has as its precursor the amino acid tryptophan [40, 41]. The enzyme arylalkylamine* N-*transferase (AANAT) is locally controlled since its transcription in the photoreceptors is regulated by a circadian clock that is independent of the hypothalamus [37, 42–44]. The AANAT encoding gene, the* AANAT* gene, contains circadian E box enhancers and AMP-response elements in its promoter, contributing to its circadian expression [45, 46]. It was shown in that studies that the chicken retina contained a TTATT repeat sequence and an analog of the canonical CRE (c-AMP-response element) to drive* AANAT *expression from its basal and c-AMP-driven promotors and these sequences are targeted by CREB, c-FOS, and Jun-D transcription factors. Also posttranslational regulation of the* AANAT *gene is subjected to circadian control [42, 43]. Acute light exposure, mediated by dopamine, induces a reduction in the c-AMP level in photoreceptors, which decreases binding of cyclic AMP-response elements in the* AANAT *promoter and stimulates dephosphorylation of AANAT and its degradation [47, 48]. Another enzyme essential for melatonin biosynthesis in the retina is tryptophan hydroxylase, converting tryptophan to 5-hydroxytryptophan (5HTP), and is encoded by the* Tph *gene, the expression of which in the retina is controlled in a circadian manner, exhibiting a similar daily rhythm to AANAT activity [49, 50].

It is not definitely proven whether the intact human retina produces its own melatonin or takes it up from the blood [51]. The melatonin-synthesis system is, however, functional in cultures of human retinal pigment epithelium cell, ARPE-19 [52]. Although melatonin receptors, MT1 and MT2, are present in the human retina, the presence of acetylserotonin methyltransferase (ASMT) is very low, so the receptors can bind circulating melatonin [53, 54]. However, ASMT and AANAT were detected in the retina of* Macaca mulatta*, but the transcript concentration of the former is very low and detected only at night [53]. In these studies mRNA levels of AANAT were constant for the entire twenty-four hours, but its activity increased at night. This suggests that melatonin in the human retina is produced not associated with the increased expression of genes involved in its biosynthesis but rather by the activation of their protein products; this suggestion is supported by the studies in human retinoblastoma cells [55]. Retinal melatonin receptors in humans are located in the rod photoreceptors and ganglion cells [54]. Melatonin influences the electroretinogram response suggesting that it can play a role in the shifting between day- and night-activity in the retina and it does so by regulating the retina clock [56–58].

Melatonin modulates the expression of retinal genes, that is,* PER1*,* PER2*, and* BMAL1, *responsible for controlling circadian rhythms in this tissue, as well as the genes regulating their expression:* DBP*,* NAMPT*, and* c-FOS* were rhythmically modulated in the mouse retina and in the photoreceptor layer [59]. However, inhibition of melatonin signaling essentially changed the pattern of expression of these genes, but only in ganglion cells, while in the photoreceptor layer only* BMAL1* expression pattern is altered. Thus, melatonin is important in the regulation of the expression of clock genes especially in the ganglion cells layer and less so in the photoreceptors. These results may seem somehow surprising as melatonin receptors are expressed in mouse photoreceptors [60]. It is likely that the expression of clock genes in photoreceptors is regulated by neurohormonal signals or the expression of* BMAL1* is not required in a rhythmic fashion [33].

## 4. Age-Related Macular Degeneration and the Role of Oxidative Stress in Its Pathogenesis

**Figure 3:**
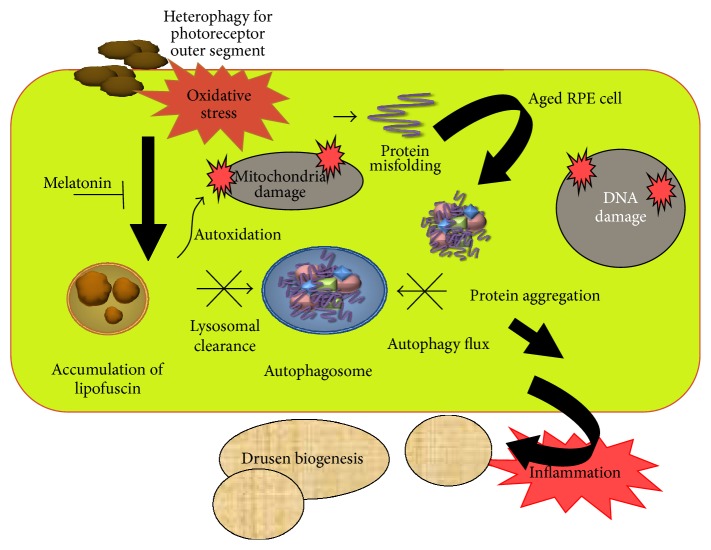
Protein aggregation in aged RPE cells is involved in AMD pathology. RPE cells are constantly exposed to oxidative stress. In daily visual cycle photoreceptor outer segments are phagocytosed by the RPE; this process is referred to as heterophagy. The cellular elements are degraded in the lysosomes. In aged RPE cells, lipofuscin accumulates in lysosomes as a result of a coincident decline of lysosomal enzyme activity and autophagy flux. Lipofuscin is an autooxidant that increases damage induced by oxidative stress in mitochondrial and nuclear DNA. Disturbed clearance and accumulated toxic compounds in RPE trigger the inflammation and explain the AMD-associated biogenesis of extracellular drusen formation. Melatonin both inhibits oxidative damage and suppresses inflammation.

**Figure 4:**
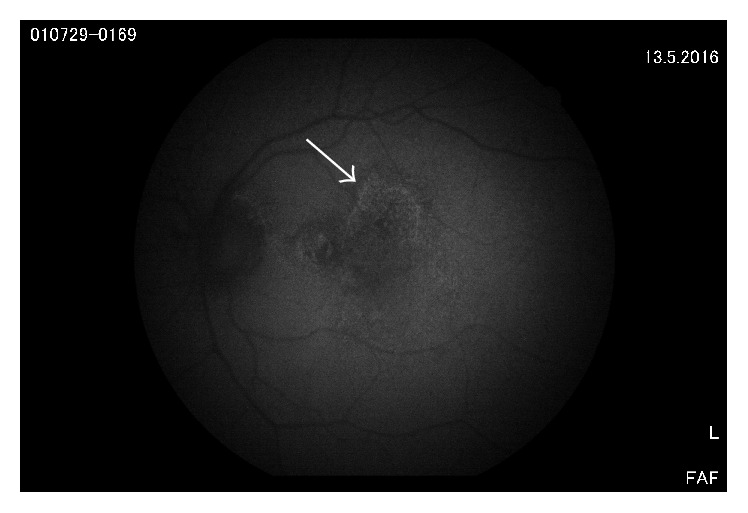
Fundus autofluorescence (FAF) from a dry AMD case. Arrow indicates the edge of lipofuscin accumulation and damage of RPE cells.

AMD is a complex eye disease, which is a serious problem in elder individuals in Western countries as it occurs in these societies with a high frequency and is a major cause of vision loss [61]. In its advanced stage AMD occurs in two forms: dry (nonexudative) and wet (exudative, neovascular). The intracellular lipofuscin and extracellular drusen accumulation within the macula region are central cellular hallmarks in the developmental process of AMD [62] (Figures 3 and 4). The environmental risk factors of AMD include advanced age, smoking, Caucasian ethnicity, hypertension, hypercholesterolemia, obesity, arteriosclerosis, female gender, lightly pigmented iris, exposure to UV and blue light, fat-rich diet, and several genetic implications [63, 64]. The majority of the environmental and some genetic risk factors are associated with oxidative stress [65, 66]. This stress is linked with an increased ROS production and can originate from many sources, including disturbed iron metabolism [67]. The retina itself is an organ highly predisposed to oxidative stress due to intense blood flow, high metabolic rate, the presence of mitochondria-rich cells, high concentrations of easily oxidizable polyunsaturated fatty acids (PUFAs) in membranes, and prolonged exposure to light. All the sources of ROS in AMD have not been identified.

Cleansing of photoreceptor outer segments in lysosomes is called heterophagy and it is a normal visual cycle regulation [68–70].

Increased autofluorescent lipofuscin accumulation can be observed in postmitotic RPE cells in the course of AMD (Figures 3 and 4). The causative association between the excess of lipofuscin and the pathogenesis of the disease is not well established. Nonetheless, this illustrates a relationship between oxidative stress and AMD and suggests that the stress can be targeted in AMD prevention and therapy. The AREDS (Age-Related Eye Disease Study) found that supplementation of the diet by antioxidants and zinc (the AREDS formulation) could reduce the risk of developing advanced AMD by 25%, although this result was subsequently questioned [71]. Our recent observation shows that nutriceuticals with omega-fatty acids and resveratrol induce lysosomal autophagy and protect RPE cells [72].

As DNA repair is a major pathway of cellular defense against oxidative stress, we studied the role of oxidative DNA damage and repair in the pathogenesis of AMD. This type of DNA damage can have various forms, but the most frequent ones are oxidative modifications to the DNA bases [73]. They are removed by base excision repair (BER) pathway, in which DNA glycosylases play a pivotal role [74]. These enzymes cut the N*-*glycosylic bond between sugar and the oxidatively modified base, leaving an apurinic or apyrimidinic (AP) site, which is then converted by an AP endonuclease into a single-strand DNA break. Some DNA glycosylases can cleave DNA strand by their AP lyase activity. The resulting DNA single-strand termini are then processed to produce 3′-OH and 5′-P ends, which can be a substrate for a DNA polymerase, assisted by auxiliary proteins. Finally, lacking phosphodiester bond is sealed by a DNA ligase. Thus, several enzymes and nonenzymatic proteins are crucial for removing oxidative DNA damage and their abnormal expression can be important in pathogenesis of many human diseases [75]. Such disturbed expression can be determined by variation in the sequence of genes encoding BER proteins. We studied several polymorphism types in the BER genes in AMD patients obtaining some correlations, which support the hypothesis related to the involvement of oxidative DNA damage and repair in AMD pathogenesis [76].

As mentioned previously, the most important genetic AMD risk factors are mutations in the genes encoding the complement factor H, with the rs1061170T>C variant identified by genomewide association studies [77]. Therefore, as oxidative stress is a major environmental risk factor, it is reasonable to investigate the role of the interaction between these two influences in AMD pathogenesis. It was suggested that chronic oxidative stress may initiate pathological changes in the retina due to damage to retinal biomolecules, resulting in chronic inflammation with the involvement of the complement system, with the pronounced role of CFH and eventually invasive immune cells contributing to retinal changes typical for AMD [66, 78] (Figure 4).

As already noted, the retina has its own circadian system, which can be important for both its physiology and pathology, implicating a potential role of melatonin in retinal physiological and pathological phenomena. The role of circadian rhythms in oxidative stress-related pathogenesis of AMD was recently reviewed by Fanjul-Moles and Lopez-Riquelme [79].

## 5. Melatonin as an Antioxidant in the Retina

Melatonin exerts its antioxidant actions by several mechanisms, including direct ROS scavenging, stimulating antioxidant enzymes, improving the functioning of mitochondrial ETC, reducing the extent of electron leakage from the mitochondrial complexes, and improving the efficacy of other antioxidants [4, 80]. The antioxidant action of melatonin can be also expressed by increasing the efficacy of removing oxidative DNA damage [81].

Melatonin exerts protective effects in several experimental models of oxidative stress-related diseases of the human eye; these are summarized in an old review by Siu et al. [82]. The diseases include photokeratitis, cataract, glaucoma, retinopathy of prematurity, and ischemia/reperfusion injury. The studies reviewed herein suggest the potential use of melatonin as a prophylaxis to protect against disturbance of visual functions, especially in the elderly. Moreover, the results justify continued research related to the molecular mechanisms of action of melatonin in the eye.

A one-minute exposure to light produced ROS in isolated and dark adapted frog photoreceptors; this process was inhibited by picomolar concentrations of melatonin, which, on the other hand, increased the prooxidant effect of the light at higher concentrations [83]. In a similar study melatonin was shown to act synergistically with vitamin E to reduce the level of nitric oxide-induced lipid peroxidation in the retina of rats [84]. Melatonin protects against lipid peroxidation induced in PUFAs in rod outer membrane segment cells [85].

Melatonin at concertation ranging from 10^−10^ to 10^−4^ protects human retinal epithelium cells in culture against cytotoxic effects of hydrogen peroxide [86]. Luzindole, a melatonin membrane-receptor antagonist, exhibited a differential modulation of the protective effect of melatonin on RPE cells, which depended on melatonin concentration. At a low, up to 10^−8^ M, concentration range, luzindole completely blocked this effect, but at concentrations above 10^−6^ M the receptor antagonist did not affect melatonin action. Therefore, it can be concluded from that study that melatonin can exert the protective effects against hydrogen peroxide-induced damage in human RPE either indirectly by the activation of melatonin receptors or directly via its antioxidant effect or by a combination of both, depending on its concentration. However, high exogenous concentrations of melatonin increase light-induced damage to photoreceptor [35].

Oxidation of xanthine or hypoxanthine is catalyzed by xanthine oxidase (XO) and produces superoxide radical and hydrogen peroxide, which can yield hydroxyl radicals [87]. XO was detected in the endothelium of retinal capillaries and some cone photoreceptors [88]. It was shown that melatonin protected cultured human retinal neurons from the detrimental action of ROS-producing XO, which also prevents the associated apoptosis and necrosis [89]. Melatonin was also shown to be effective against lipid peroxidation, assessed by the content of malondialdehyde in rat retinal homogenates [90].

Hydrogen peroxide can induce a short-term oxidative stress, which leads to oxidative DNA damage. As mitochondria are a generous source of ROS, mitochondrial DNA (mtDNA) is a prime candidate to ROS-induced damage. Melatonin was shown to be effective in the protection of mtDNA of ARPE-19 cells against hydrogen peroxide-induced damage [91]. In that study, melatonin did not exhibit a protective effect against cytotoxicity induced by H_2_O_2_. Another major source of free radical production is ischemia/reperfusion. In a single study, melatonin was found not to protect the retina from ischemia/reperfusion injury [92].

Glaucoma, a major cause of loss of vision worldwide, is mostly characterized by increased intraocular pressure (IOP). IOP underlies the degeneration of retinal ganglion cells and damage to optic nerve head; oxidative stress may be important for its pathology [93, 94]. A significant decrease in retinal antioxidant defense was observed in a rat model mimicking human open-angle glaucoma and since melatonin ameliorated this process, the indoleamine may be a useful component of a therapeutic strategy in glaucoma [95]. This is also justified by a hypotensive effect of melatonin resulting in a reduction of IOP in a mouse glaucoma model [96].

Retinopathy is a major complication of type 2 diabetes mellitus and oxidative stress is considered as an important pathogenic factor in this disease [97]. The retina of streptozotocin-induced diabetic rat was reported to have depleted glutathione and downregulation of the enzyme critical for its production, glutamate cysteine ligase [98]. Melatonin upregulated the enzyme by the maintaining of Nrf2, a major transcription factor regulating the expression of antioxidant proteins. In the nucleus Nrf2 stimulates the phosphorylation of AKT, a protein critical for DNA damage signaling. Moreover, this study showed that melatonin inhibits some proinflammatory molecules through the nuclear factor-kappa B (NF-*κ*B) pathway, suggesting that its antioxidative action in the retina is not limited to a direct interaction with components of antioxidative system and ROS scavenging.

## 6. Melatonin in AMD

AMD is an age-related neurodegenerative disease and several studies reported that melatonin delayed the neurodegenerative processes associated with aging [99]. If melatonin is involved in the modulation of many important retinal functions, as indicated above, and if it inhibits age-related changes in the retina, it is justified to assume its potential to influence the onset and progression of AMD. This is supported by the results showing that the daily rhythms in the electroretinogram responses and scotopic threshold response, which is a dark adapted response of proximal retina, are affected by age and are associated with a decrease in melatonin synthesis [100]. These two effects can be considered as early markers preceding degenerative age-related changes in the retinal structure and function, including those typical for AMD. These effects were not rescued by exogenous melatonin, which suggests that they could be related to a progressive loss of the sensitivity of melatonin receptors which may occur in advanced age. It has been speculated that complete insensitivity of melatonin receptors is possible in a certain age. In a study, in which 55 patients with either dry or wet AMD take 3 mg of melatonin before bedtime each day for at least 3 months, their visual acuity was stabilized [101].

In the context of the role of melatonin in AMD, several reports suggest a decrease in melatonin synthesis with age [99]. This is another rationale for considering this indole as a player in AMD pathogenesis. However, it is not certain whether such a tendency is typical for retinal melatonin, but it was observed that the level of ANAAT decreased with age [102, 103]. It was reported that the amount of 6-sulphatoxymelatonin in nocturnal urine was lower in AMD patients than in age-matched controls [104].

As mentioned, antioxidative enzymes are stimulated by the Nrf2-dependent signaling and aryl hydrocarbon receptor (AhR) is a transcription factor regulating the expression of enzymes involved in the metabolism of xenobiotics, which can produce prooxidants [105]. On the other hand, some genes regulated by AhR are indirectly involved in antioxidant defense, so both types of proteins are important for oxidative stress in the cell [106]. Thus, the interaction of melatonin with the pathways of both kinds of enzymes may be important for the pathogenesis of oxidative stress-related diseases, including AMD. Using the premature aging OXYS rats, as a model for human AMD, it was shown that melatonin downregulated the expression of the cytochrome P* CYP1A2* and* CYP1B1* genes and exerted no effect of the Nrf2-regulated genes in the retina of these rats [107].

It has been hypothesized that AMD is initiated by aged and damaged RPE cells [108] (Figure 3). It also was shown that the process of telomere shortening, critical for senescence, could be dependent on environmental oxidative stress and the activity of antioxidant enzymes [109]. Therefore, it is justified to examine the possible effects of melatonin on the senescence of RPE cells in AMD patients. Moreover, it was hypothesized that melatonin can stimulate the activity of telomerase to rebuild the telomeres in senescent RPE cells in AMD retinas, which are important for both prevention and therapy of AMD [110]. The retina contains potentially active telomerase, which can be implicated in functions other than telomere maintenance functions, including expression of retinal cell phenotypes [111]. Overexpression of the catalytic subunit of the enzyme was observed in a mouse model of retinopathy [112]. A high expression of telomerase observed in retinoblastoma is not surprising as it is considered as a major mechanism of cancer cell immortality [113, 114]. Melatonin modulates the expression and activity of telomerase [3].

Retinal hypoxia in AMD results in the breakdown of the inner retina-blood-barrier while melatonin protects against this change [115, 116]. The mechanism of this effect can include the interaction of melatonin with vascular endothelial growth factor (VEGF), as melatonin was reported to decrease the level of VEGF in hypoxia in the choroid plexus, which may be crucial in preventing the development of exudative form of AMD [117]. As anti-VEGF strategy is routinely used in the therapy of exudative AMD, it can be anticipated whether melatonin supplementation might decrease the number of intravitreal injections [118, 119].

Blue light is used to send information to synchronize internal biological rhythm with changes in the environment and melatonin is involved in this process [120]. However, these wavelengths are also an AMD pathogenesis factor [16]. This justifies the randomized controlled trial, called the CLOCK-IOL color study, on the effect of intraocular lenses blocking blue light on circadian rhythmicity which has been set [121]. However, it should be taken into account that both the involvement of environmental exposure to light in AMD pathogenesis and the protective role of blue-blocking intraocular lenses in AMD have been questioned [122].

Impaired autophagy has been associated with AMD which is consistent with the accumulation of lipofuscin and extracellular drusen [123, 124] (Figure 3). Interestingly, melatonin was shown to influence autophagy in several studies [125, 126]. Therefore, melatonin reduces deregulated autophagy in AMD and inhibits progression of the disease.

It is intriguing that AMD affects only a small part of the retina, that is, the macula. The macula comprises only 2.1% of the retina; when it undergoes degeneration the remainder of the retina remains unaffected. The reason for this is not completely clear, but several potential explanations can be considered. The central retina, including the macula, is substantially thicker than peripheral retina, which is especially seen in the relative thickness of ganglion cell layers, inner plexiform layers, and nerve fiber layer; the increased thickness is due to larger number and higher packaging density of ganglion cells in the central compared to the peripheral retina. As a consequence, cells in the central retina may be more susceptible to chemical or physical factors important for AMD pathogenesis. As mentioned above, the retina is characterized by an intense blood supply, but this feature also differs in the central and peripheral retina. The blood supply to the avascular macula depends exclusively on the choroidal circulation, while the vascularized peripheral retina relies on both the central retinal and choroidal blood vessels. Therefore, degeneration of RPE cells may be more severe for the central retina cells, whilst the peripheral retina cells can survive the consequences of RPE degeneration as its blood supply is more abundant. Moreover, melatonin receptors are located on the rod photoreceptors, which are mainly in the periphery; this could be a factor in determining the location of ADM. Finally, if we assume that melatonin acts in the retina as an antioxidant, its uptake from the retinal blood vessels in the periphery may be more efficient than in the macula. This difference could be, at least in part, responsible for higher susceptibility of the central retina to oxidative stress following RPE damage. In reference to degenerative conditions of the retina, the location of retinitis pigmentosa (RP), an eye disease affecting predominantly the peripheral retina, thus topographically different from AMD, is intriguing. The issue of the significance of melatonin in RP clearly has not been adequately addressed.

## 7. Conclusion and Perspectives

It is important to have a further insight into differential involvement of melatonin in regulation of circadian genes in photoreceptors and ganglion cells. This can lead to the identification of cells in the inner and ganglion layers affected by melatonin signaling.

As we mentioned in the Introduction, retinal cells, including RPE cells, are rich in mitochondria and these organelles are a major source of ROS, which are natural products of the functioning of the mitochondrial ETC. These ROS play a significant role in AMD pathogenesis. However, chronic oxidative stress in RPE cells can influence the dynamics of the mitochondrial network, that is, the ratio of mitochondrial association (fusion) and disassociation (fission) [127, 128]. Consequently, mitochondria are both a source and a target of ROS in RPE cells and are likely involved in AMD pathogenesis; this is supported by the results of a large variety of publications [129–131]. Postmortem studies of eyes affected by AMD revealed excess damage to mitochondrial DNA, which is a result of oxidative stress [132, 133]. Therefore, melatonin involvement in the mitochondrial control could be addressed in AMD. The association between melatonin and mitochondria is supported by the prediction that mitochondria may be primary sites for melatonin synthesis [134]. Moreover, melatonin was shown to behave as a synthetic, mitochondria-targeted antioxidant; it is reported to be in high concentrations in this organelle [80].

The activity of telomerase in the retina is especially intriguing, as this enzyme is usually activated in cancer cells [135]. The involvement of telomerase in the maintenance of telomere length in retinal cells and influence of oxidative stress and melatonin in this process deserve further attention as telomere shortening is associated not only with normal aging, but also with premature aging and age-related diseases [136, 137]. It should be tempting to develop this topic relative to AMD therapy. The usefulness of cell and gene therapy targeted to telomerase was evaluated [138]; it seems that melatonin targeting this enzyme in the retina could be a direct strategy to prevent AMD. The role of telomerase in oxidative stress associated with AMD is supported by the observation that the activity of this enzyme in human retinal progenitor cells was sensitive to oxygen concentration [139]. It was also observed that ectopic expression of telomerase extended the life span of human retinal microvascular cell line [140].

The antioxidant effect of melatonin, which would likely be crucial for its protective role in AMD, can be broadly divided into receptor-dependent and receptor-independent processes. Although the latter can be attributed to radical-scavenging properties of melatonin, the former needs further research. The role of melatonin in the pathogenesis of ocular diseases is supported by the basic fact that the concentration of this indole in ocular aqueous humors (10^−9^–10^−8^) can be higher than in the serum (2 × 10^−10^–10^−9^) [141, 142].

There are too few clinical trials with melatonin in AMD patients to establish a reliable relationship between the level of this indole and AMD occurrence/progression, but some data suggest a reduced level of melatonin in AMD [86, 104]. Since melatonin generally lacks significant toxicity, a clinical study with melatonin supplementation for AMD prevention and treatment should be considered. Experience coming from other clinical trials with melatonin suggests that daily supplementation with melatonin could be sufficient to check clinical effectiveness of melatonin in AMD [86].
